# Supplementary material for: De novo E-cadherin/catenin complex formation controls basal epithelial mechanics and force transmission for apoptotic cell clearance
Source: Nat Commun. 2026 Aug 27;17:8900. doi: 10.1038/s41467-026-76710-1 (PMC13522565; doi:10.1038/s41467-026-76710-1)
Supplement: Supplementary file 2 [file 41467_2026_76710_MOESM2_ESM.pdf]

Description of Additional Supplementary Files

File name: Supplementary Movie 1
Description: Time-lapse SIM imaging of the basal and apical side of the enveloping layer (EVL) in a zebrafish embryo at ~30% epiboly, expressing Lifeact-GFP to visualize filamentous actin. The movie shows dynamic actin flow patterns at the basal surface of epithelial cells versus a less dynamic actin organization at the apical cortex. Images were generated as maximum-intensity projections of z-stacks (~4.4 μm thickness, basal side) and (~0.4 μm thickness, apical side). Scale bars: 10 μm.

File name: Supplementary Movie 2
Description: Phagocytic clearance in a control embryo (left) and E-cadherin morpholino injected embryo (Ecad MO, right) expressing Lifeact-GFP (cyan). Apoptotic targets (Bax+ cells) express a plasma membrane marker (red, Lyn-tdTomato). The phagocytic uptake of apoptotic targets by the embryonic surface epithelium (upper panel, z = 0 μm) and the phagocytic clearance dynamics in the embryo interior underneath the epithelium in a control (z = -14 μm) and an Ecad MO (z = -10 μm) embryo are shown. Embryos were imaged for 2 hours from 30% epiboly (4.7 hpf) to shield stage (6.5 hpf). Embryos were obtained from the Tg(actb2:Lifeact-GFP) line. Time is indicated in h:min:s:ms. Scale bar: 50 μm.

File name: Supplementary Movie 3
Description: Phagocytic uptake events of apoptotic targets by single epithelial cells in an embryo expressing Lifeact-GFP (cyan). Apoptotic targets (Bax+ cells) express a plasma membrane marker (red, Lyn-tdTomato). Lines indicate the boundaries of the epithelial cell before (white) and just after (yellow) an apoptotic uptake event. The embryo was imaged from dome stage (4.5 hpf) to shield stage (6.5 hpf) and was obtained from the Tg(actb2:Lifeact-GFP) line. Time is indicated in min. Scale bar: 20 μm.

File name: Supplementary Movie 4
Description: E-cadherin localization at two independent phagocytic cups (arrowheads) during the clearance of two apoptotic targets (red, Bax+ cells co-expressing the plasma membrane (PM) marker Lyn-tdTomato). The embryo was obtained from the CRISPR/Cas9 knock-in line KI(mlanYFP)^{xt17}cdh1-YFP expressing E-cadherin-YFP (cyan) at endogenous levels. Time is indicated in min. Scale bar: 20 μm.

File name: Supplementary Movie 5
Description: Transient E-cadherin enrichment (arrowhead) in a newly formed phagosome upon uptake of an apoptotic cell (red, Bax+ cells co-expressing the plasma membrane (PM) marker Lyn-tdTomato). The embryo was obtained from the CRISPR/Cas9 knock-in line KI(mlanYFP)^{xt17}cdh1-YFP expressing E-cadherin-YFP (cyan) at endogenous levels. Time is indicated in min. Scale bar: 20 μm.

File name: Supplementary Movie 6
Description: E-cadherin localization at an "epithelial arm" protrusion associated with an apoptotic target (red, Bax+ cells co-expressing the plasma membrane (PM) marker Lyn-tdTomato). The embryo was obtained from the CRISPR/Cas9 knock-in line KI(mlanYFP)^{xt17}cdh1-YFP expressing E-cadherin-YFP (cyan) at endogenous levels. Time is indicated in min. Scale bar: 20 µm.

File name: Supplementary Movie 7
Description: Uptake of apoptotic targets and phagosome formation over time by single epithelial cells in a control (upper panel) and an E-cadherin morpholino (Ecad MO) injected embryo (lower panel) expressing Lifeact-GFP (cyan). Apoptotic targets (Bax+ cells) express a plasma membrane marker (red, Lyn-tdTomato). White lines indicate the boundaries of the same epithelial cell in control and Ecad MO embryos at the start and end point of the movie. White arrowheads indicate individual uptake events and the formation of a new phagosome. Embryos were imaged for 2 hours from 30% epiboly (4.7 hpf) to shield stage (6.5 hpf). Embryos were obtained from the Tg(actb2:Lifeact-GFP) line. Time is indicated in h:min:s:ms. Scale bar: 20 µm.

File name: Supplementary Movie 8
Description: Epithelial tissue and single cell F-actin dynamics (cyan) in the presence of apoptotic cells (red, Bax+ cells co-expressing the plasma membrane marker Lyn-tdTomato) in control (left) and E-cadherin MO injected (right) embryos. Embryos were obtained from the Tg(Krt18:Gal4FF/UAS:Lifeact-GFP) line, which expressed Lifeact-GFP exclusively in the epithelial tissue. Time is indicated in h:min:s. Scale bar: 20 µm.

File name: Supplementary Movie 9
Description: In vivo tracking (yellow lines) of apoptotic targets (red, Bax+ cells co-expressing the plasma membrane marker Lyn-tdTomato) in a control embryo (left) and an E-cadherin deficient embryo (Ecad MO, right) obtained from the Tg(actb2:Lifeact-GFP) line. Time is indicated in h:min:s. Scale bar: 50 µm.

File name: Supplementary Movie 10
Description: Phagocytic clearance in a control (left) and a DECMA-1 injected embryos (right) expressing Lifeact-GFP (cyan). Apoptotic targets (Bax+ cells) express a plasma membrane marker (red, Lyn-tdTomato). The phagocytic uptake of apoptotic targets by the embryo epithelium (upper panel, z = 0 µm) and phagocytic clearance dynamics in the embryo interior underneath the epithelium in a control (z = -8 µm) and a DECMA-1 injected embryo (z = -14 µm) are shown. Embryos were imaged for 2 hours from 30% epiboly (4.7 hpf) to shield stage (6.5 hpf). Embryos were obtained from the Tg(actb2:Lifeact-GFP) line. Time is indicated in h:min:s:ms. Scale bar: 50 µm.

File name: Supplementary Movie 11
Description: Phagocytic clearance in control (left) and DECMA-1 injected embryos (right) expressing Lifeact-GFP after apoptotic cell fragmentation occurred at 50% epiboly stage (5.5

hpf). Apoptotic targets express a plasma membrane marker (red, Lyn-tdTomato). The upper panel shows a maximum-intensity projection of the embryonic epithelium and the location of phagosomes before DECMA-1 injection and a small amount of newly formed phagosomes after 2 hours of imaging (both indicated by white arrowheads). The lower panel shows the embryo interior (z = -10 μm) underneath the epithelium, supporting the availability of small apoptotic targets (yellow arrowheads) in the embryo interior. Embryos were imaged for 2 hours from 30% epiboly (4.7 hpf) to shield stage (6.5 hpf). Embryos were obtained from the Tg(actb2:Lifeact-GFP) line. Time is indicated in h:min:s:ms. Scale bar: 50 μm.

File name: Supplementary Movie 12
Description: Phagocytic clearance of transplanted apoptotic cells from a control donor embryo (left) or a donor embryo injected with E-cadherin morpholino (Ecad MO, right) into a host embryo expressing Lifeact-GFP (cyan). Apoptotic targets (Bax+ cells) express a plasma membrane marker (red, Lyn-tdTomato). The upper panel shows the phagocytic uptake of apoptotic targets by the embryo epithelium. The lower panel shows the phagocytic clearance dynamics in the embryo interior for control apoptotic cells (z = -16 μm) and apoptotic cells obtained from an Ecad MO donor embryo (z = -12 μm). Embryos were imaged for 2 hours from 30% epiboly (4.7 hpf) to shield stage (6.5 hpf). Embryos were obtained from the Tg(actb2:Lifeact-GFP) line. Time is indicated in h:min:s:ms. Scale bar: 50 μm.

File name: Supplementary Movie 13
Description: Phagocytic clearance of transplanted PS+ lipid aggregates (red, synthetic apoptotic targets) in a control embryo (left) and an E-cadherin morphant embryo (Ecad MO, right) expressing Lifeact-GFP (cyan). The phagocytic uptake of synthetic apoptotic targets by the embryonic epithelium (upper panel) and the phagocytic clearance dynamics in the embryo interior (lower panel) are shown. Embryos were imaged over a duration of 3 hours from 30% epiboly (4.7 hpf) to 75% epiboly stage (8 hpf). Embryos were obtained from the Tg(actb2:Lifeact-GFP) line. Time is indicated in h:min:s. Scale bar: 40 μm.

File name: Supplementary Movie 14
Description: E-cadherin localization at a phagocytic cup (arrowheads) during the clearance of a lipid aggregate (red) from the embryo interior (bottom) to the epithelium (top). Embryo was obtained from the CRISPR/Cas9 knock-in line KI(mlanYFP)xt17cdh1-YFP expressing E-cadherin-YFP (cyan) at endogenous levels. Time is indicated in min. Scale bar: 20 μm.

File name: Supplementary Movie 15
Description: Localization of α-catenin-mCherry (white arrowheads, left) during the uptake of an apoptotic cell target (Bax+ cells co-expressing the PM marker Lyn-Cerulean, asterisks, right) by epithelial cells. The upper panel shows a top view and the bottom panel shows a transversal view of epithelial cells. White asterisks indicate the apoptotic cell targets within the embryo interior, while the yellow asterisk mark the engulfed phagosome. Time is indicated in h:min:s. Scale bar 10 μm.

File name: Supplementary Movie 16

Description: Localization of β-catenin-GFP (white arrowheads, left) during the uptake of an apoptotic cell target (Bax+ cells co-expressing the PM marker Lyn-tdTomato, asterisks, right) by epithelial cells. The upper panel shows a top view and the bottom panel shows a transversal view of epithelial cells. White asterisks indicate the apoptotic cell target within the embryo interior, while yellow asterisk marks the engulfed phagosome. Time is indicated in h:min:s. Scale bar 10 μm.

File name: Supplementary Movie 17

Description: Phagocytic clearance of apoptotic targets (Bax+ cells co-expressing the PM marker Lyn-tdTomato, red) in control (left) and α-catenin morphant (MO, right) embryos expressing Lifeact-GFP (cyan). The upper panel shows the phagocytic uptake of apoptotic targets by the embryonic epithelium. The lower panel shows the phagocytic clearance dynamics within the embryo interior. Embryos were imaged for 2 hours from 30% epiboly (4.7 hpf) to shield stage (6.5 hpf) and were obtained from the Tg(actb2:Lifeact-GFP) line. Time is indicated in h:min:s. Scale bar 40 μm.

File name: Supplementary Movie 18

Description: In vivo tracking (yellow lines) of apoptotic targets (red, Bax+ cells co-expressing the plasma membrane marker Lyn-tdTomato) in a control (left) and an α-catenin deficient embryo (α-catenin MO, right) obtained from the Tg(actb2:Lifeact-GFP) line. Time is indicated in h:min:s. Scale bar: 40 μm.

File name: Supplementary Movie 19

Description: Phagocytic clearance of apoptotic targets (co-expressing the PM marker Lyn-tdTomato, red) in control (left) and α-cateninΔABS overexpressing (right) embryos expressing Lifeact-GFP (cyan). The upper panel shows the phagocytic uptake of apoptotic targets by the embryo epithelium. The lower panel shows phagocytic clearance dynamics within the embryo interior. Embryos were imaged for 3 hours from 30% epiboly (4.7 hpf) to 75% epiboly (8 hpf) and were obtained from the Tg(actb2:Lifeact-GFP) line. Time is indicated in h:min:s. Scale bar: 40 μm.

File name: Supplementary Movie 20

Description: Transient accumulation of Myosin II during the uptake of an apoptotic cell fragment (co-expressing the PM marker Lyn-tdTomato, grey) by epithelial cells. The upper panel shows a top view and the bottom panel shows a transversal view of epithelial cells. Embryos were obtained from the Tg(actb2:myl12.1-EGFP) line and imaged at 50% epiboly (5 hpf). Arrowheads indicate a local enrichment of Myosin II during the uptake of an apoptotic cell. Time is indicated in h:min:s. Scale bars: x: 10 μm, z: 5 μm.

File name: Supplementary Movie 21

Description: Localization of Myosin II at the apical domain and the basal epithelial surface (middle) at the contact sites with apoptotic cells (right) in control embryos. Apoptotic targets (apoptotic Bax+ cells) express a plasma membrane marker (grey, Lyn-tdTomato).

Arrowheads indicate the transient accumulation of Myosin II during phagocytic uptake, while asterisks indicate the resulting phagosomes. Embryos were imaged for 1 hour, from 30% epiboly (4.7 hpf) to 50% epiboly stage (5.5 hpf). Embryos were obtained from the Tg(actb2:myl12.1-EGFP) line. Time is indicated in h:min:s. Scale bar: 20 μm.

File name: Supplementary Movie 22
Description: Localization of Myosin II at the apical domain (left) versus its accumulation on the basal epithelial surface (middle) at contact sites with apoptotic cells (right) in E-cadherin morphant (MO) embryos. Apoptotic targets (apoptotic Bax+ cells) express a plasma membrane marker (grey, Lyn-tdTomato). Arrowheads indicate sites of local Myosin II accumulation in epithelial cells, corresponding to contact sites with apoptotic targets. Embryos were imaged for 1h, from 30% epiboly (4.7 hpf) to 50% epiboly stage (5.5 hpf). Embryos were obtained from the Tg(actb2:myl12.1-EGFP) line. Time is indicated in h:min:s. Scale bar: 20 μm.

File name: Supplementary Movie 23
Description: Phagocytic clearance of apoptotic targets (co-expressing the PM marker Lyn-tdTomato, red) in E-cadherin morphant (MO, left) and E-cadherin MO embryos overexpressing the Myosin phosphatase (MP) complex (right), both embryos ubiquitously expressing Lifeact-GFP (cyan). The upper panel shows the phagocytic uptake of apoptotic targets by the embryo epithelium. The lower panel shows the phagocytic clearance dynamics within the embryo interior. Embryos were imaged for 3 hours from 30% epiboly (4.7 hpf) to 75% epiboly (8 hpf) and were obtained from the Tg(actb2:Lifeact-GFP) line. Time is indicated in h:min:s. Scale bar: 40 μm.

File name: Supplementary Movie 24
Description: Localization of GFP-p120-catenin during the uptake of an apoptotic cell fragment (co-expressing the PM marker Lyn-Cerulean, grey) by an epithelial cell. The upper panel shows a top view and the bottom panel shows a transversal view of epithelial cells. Embryos were obtained from the Tg(actb2:Lyn-tdTomato) line injected with GFP-p120-catenin and imaged at 50% epiboly (5 hpf). Arrowheads indicate the local enrichment of GFP-p120-catenin during the uptake of an apoptotic cell fragment. Time is indicated in h:min:s. Scale bars: x: 10 μm, z: 5 μm.

File name: Supplementary Movie 25
Description: Localization of Myosin II at the apical domain (left) versus its accumulation on the basal epithelial surface (middle) at the contact sites with apoptotic cells (right) in p120-catenin MO embryos. Apoptotic targets (apoptotic Bax+ cells) express a plasma membrane marker (grey, Lyn-tdTomato). Arrowheads indicate sites of local Myosin II accumulation corresponding to epithelial interaction sites with apoptotic targets. Embryos were imaged for 1 hour from 30% epiboly (4.7 hpf) to 50% epiboly stage (5.5 hpf). Embryos were obtained from the Tg(actb2:myl12.1-EGFP) line. Time is indicated in h:min:s. Scale bar: 20 μm.

File name: Supplementary Movie 26

Description: Phagocytic clearance in a control embryo (left) and a p120-catenin morpholino injected embryo (right) expressing Lifeact-GFP (cyan). Apoptotic targets (Bax+ cells) express a plasma membrane marker (red, Lyn-tdTomato). The phagocytic uptake of apoptotic targets by the embryo epithelium (upper panel) and the phagocytic clearance dynamics in the embryo interior underneath the epithelium for a control embryo (z = -12 μm) and a p120-catenin MO embryo (z = -6 μm) are shown. Embryos were imaged for 2 hours from 30% epiboly (4.7 hpf) to shield stage (6.5 hpf). Embryos were obtained from the Tg(actb2:Lifeact-GFP) line. Time is indicated in h:min:s:ms. Scale bar: 50 μm.

File name: Supplementary Movie 27

Description: Localization of Myosin II at the apical domain (top) and the basal epithelial surface (middle) at the contact sites with apoptotic cells (Bax+ cells co-expressing the PM marker Lyn-tdTomato, right) in α-catenin MO embryos. Embryos were imaged for 40 min starting from 30% epiboly (4.7 hpf). Embryos were obtained from the Tg(actb2:myl12.1-EGFP) line. Time is indicated in h:min:s. Scale bar: 20 μm.
