# Supplementary material for: De novo E-cadherin/catenin complex formation controls basal epithelial mechanics and force transmission for apoptotic cell clearance
Source: Nat Commun. 2026 Aug 27;17:8900. doi: 10.1038/s41467-026-76710-1 (PMC13522565; doi:10.1038/s41467-026-76710-1)
Supplement: Supplementary file 3 [file 41467_2026_76710_MOESM30_ESM.pdf]

nature portfolio

Corresponding author(s): Verena Ruprecht

Last updated by author(s): Jul 7, 2026

Reporting Summary

Nature Portfolio wishes to improve the reproducibility of the work that we publish. This form provides structure for consistency and transparency in reporting. For further information on Nature Portfolio policies, see our Editorial Policies and the Editorial Policy Checklist.

Statistics

For all statistical analyses, confirm that the following items are present in the figure legend, table legend, main text, or Methods section.

n/a	Confirmed	
☐	☒	The exact sample size (*n*) for each experimental group/condition, given as a discrete number and unit of measurement
☐	☒	A statement on whether measurements were taken from distinct samples or whether the same sample was measured repeatedly
☐	☒	The statistical test(s) used AND whether they are one- or two-sided<br>*Only common tests should be described solely by name; describe more complex techniques in the Methods section.*
☒	☐	A description of all covariates tested
☐	☒	A description of any assumptions or corrections, such as tests of normality and adjustment for multiple comparisons
☐	☒	A full description of the statistical parameters including central tendency (e.g. means) or other basic estimates (e.g. regression coefficient) AND variation (e.g. standard deviation) or associated estimates of uncertainty (e.g. confidence intervals)
☐	☒	For null hypothesis testing, the test statistic (e.g. *F*, *t*, *r*) with confidence intervals, effect sizes, degrees of freedom and *P* value noted<br>*Give P values as exact values whenever suitable.*
☒	☐	For Bayesian analysis, information on the choice of priors and Markov chain Monte Carlo settings
☒	☐	For hierarchical and complex designs, identification of the appropriate level for tests and full reporting of outcomes
☒	☐	Estimates of effect sizes (e.g. Cohen's *d*, Pearson's *r*), indicating how they were calculated

Our web collection on statistics for biologists contains articles on many of the points above.

Software and code

Policy information about availability of computer code

Data collection	Microscopy image acquisition software: Las X software from Laica version LAS X 3.5.7.23225 and ZEN Black software from ZEISS.
Data analysis	Image analysis was performed with FIIJI and MATLAB. Statistical tests were performed using R and GraphPad Prism version 10.1.0. (for Windows and for Mac, GraphPad Software, Boston, Massachusetts, UAS, www.graphpad.com). Data plots were generated using Python scripts (Python 3 reference manual10) and GraphPad Prism.

For manuscripts utilizing custom algorithms or software that are central to the research but not yet described in published literature, software must be made available to editors and reviewers. We strongly encourage code deposition in a community repository (e.g. GitHub). See the Nature Portfolio guidelines for submitting code & software for further information.

Data

Policy information about availability of data

All manuscripts must include a data availability statement. This statement should provide the following information, where applicable:

- Accession codes, unique identifiers, or web links for publicly available datasets
- A description of any restrictions on data availability
- For clinical datasets or third party data, please ensure that the statement adheres to our policy

Imaging data are made available on request.

Research involving human participants, their data, or biological material

Policy information about studies with human participants or human data. See also policy information about sex, gender (identity/presentation), and sexual orientation and race, ethnicity and racism.

Reporting on sex and gender	Not applicable.
Reporting on race, ethnicity, or other socially relevant groupings	Not applicable.
Population characteristics	Not applicable.
Recruitment	Not applicable.
Ethics oversight	Not applicable.

Note that full information on the approval of the study protocol must also be provided in the manuscript.

Field-specific reporting

Please select the one below that is the best fit for your research. If you are not sure, read the appropriate sections before making your selection.

☒ Life sciences ☐ Behavioural & social sciences ☐ Ecological, evolutionary & environmental sciences

For a reference copy of the document with all sections, see nature.com/documents/nr-reporting-summary-flat.pdf

Life sciences study design

All studies must disclose on these points even when the disclosure is negative.

Sample size	Sample sizes was determined by the number of embryos available for live cell imaging per independent experiment. All experiments have 3 replicates unless stated otherwise. In rare cases, fewer replicates were used due to sample limitations. Sample sizes of analyzed cells refer to a randomly selected sub-set of cells visible in a chosen field of view during in vivo image acquisition.
Data exclusions	All data were included in the analysis at the embryo and cell level.
Replication	Results of this study were successfully reproduced by independent researchers involved in this project.
Randomization	Parental couples of zebrafish were randomly selected from different tanks for independent experiments. Embryos from these parental couples were then randomly selected from an egg clutch for further experiments in the presented study.
Blinding	No explicit blinding was performed in the study. Data were acquired and analyzed by multiple experimentalists to support the reproducibility of results.

Reporting for specific materials, systems and methods

We require information from authors about some types of materials, experimental systems and methods used in many studies. Here, indicate whether each material, system or method listed is relevant to your study. If you are not sure if a list item applies to your research, read the appropriate section before selecting a response.

Materials & experimental systems

n/a	Involved in the study	
☐	☒	Antibodies
☐	☒	Eukaryotic cell lines
☒	☐	Palaeontology and archaeology
☐	☒	Animals and other organisms
☒	☐	Clinical data
☒	☐	Dual use research of concern
☒	☐	Plants

Methods

n/a	Involved in the study	
☒	☐	ChIP-seq
☒	☐	Flow cytometry
☒	☐	MRI-based neuroimaging

Antibodies

Antibodies used	Mouse Anti-E-Cadherin (BD Biosciences, Catalog: 610181, Clone name: 36/E-Cadherin)

Antibodies used	PKC ζ (H-1) (Santa Cruz, Catalog: sc-17781, Clone name: H-1)<br>Goat anti-Mouse IgG (H+L) Cross-Adsorbed Secondary Antibody, Alexa Fluor™ 488 (Invitrogen, Catalog: A-11001, Polyclonal)<br>p120 Antibody (6H11) (Santa Cruz, Catalog: sc-23873, Clone name: 6H11)<br>Monoclonal Anti-α-Tubulin antibody produced in mouse (Sigma-aldrich, Catalog: T9026, Clone name: DM1A)<br>Goat Anti-Mouse IgG H&L (HRP) (Abcam, Catalog: ab205719, polyclonal antibody)<br>InVivoMAb anti-mouse E-Cadherin CD324 (BioXcell, Catalog: #BE0352, Clone name: DECMA-1)<br>Goat Anti-Rat IgG H&L - Alexa Fluor® 647 (abcam, catalog: ab150167, polyclonal antibody)
Validation	*Describe the validation of each primary antibody for the species and application, noting any validation statements on the manufacturer's website, relevant citations, antibody profiles in online databases, or data provided in the manuscript.*

Eukaryotic cell lines

Policy information about cell lines and Sex and Gender in Research

Cell line source(s)	dual-tagged mouse embryonic stem cells (R1 H2B–mCherry/GFP–GPI) generated and gifted by Hadjantonakis lab (doi: 10.1002/dvg.20500)
Authentication	cell lines were not authenticated
Mycoplasma contamination	Cell lines tested negative for Mycoplasma contamination by routinary PCR testing.
Commonly misidentified lines<br>(See ICLAC register)	*Name any commonly misidentified cell lines used in the study and provide a rationale for their use.*

Animals and other research organisms

Policy information about studies involving animals; ARRIVE guidelines recommended for reporting animal research, and Sex and Gender in Research

Laboratory animals	The following zebrafish lines were used in this study: AB wild type, Tg(actb2:Lifeact-GFP), Tg(actb2:Lyn-TdTomato), Tg(actb2:myl12.1-EGFP) and KI(mlanYFP)xt17cdh1-YFP.<br>The following mouse lines were used in this study: B6CBAF1/J and CD1.
Wild animals	Not applicable.
Reporting on sex	Zebrafish and mouse embryos were used in this study and the sex of embryos is not yet determined during the developmental stage studied in this work (Zebrafish embryos: 0-10 hours post fertilization; mouse embryos: 3.5 days post coitum).
Field-collected samples	Not applicable.
Ethics oversight	Zebrafish were maintained at the aquatic facility of the Parc de Recerca Biomèdica de Barcelona (PRBB) following the standard procedures proved by the Institutional Animal Care and Use Ethic Committee (PRBB–IACUEC, CEEA-OH-PRBB:VRU-23-0014) according to national and European regulations.<br>All protocols for mouse embryo experiments were performed in coordination with the CRG Tissue Engineering Facility and were approved by the Institutional Animal Care and Use Ethic Committee (PRBB–IACUEC), conforming to the guidelines of the European Community Directive and Spanish legislation for the experimental use of animals. Mouse lines were maintained and bred according to the standard procedures at the rodent facility of the Parc de Recerca Biomèdica de Barcelona (PRBB).

Note that full information on the approval of the study protocol must also be provided in the manuscript.

Plants

Seed stocks	Not applicable.
Novel plant genotypes	Not applicable.
Authentication	Not applicable.
