# Supplementary material for: De novo E-cadherin/catenin complex formation controls basal epithelial mechanics and force transmission for apoptotic cell clearance
Source: Nat Commun. 2026 Aug 27;17:8900. doi: 10.1038/s41467-026-76710-1 (PMC13522565; doi:10.1038/s41467-026-76710-1)
Supplement: Supplementary file 4 [file 41467_2026_76710_MOESM31_ESM.pdf]

nature portfolio

Peer Review File

De novo E-cadherin/catenin complex formation controls basal epithelial mechanics and force transmission for apoptotic cell clearance

Corresponding Author: Dr Verena Ruprecht

This manuscript has been previously reviewed at another journal. This document only contains information relating to versions considered at Nature Communications.

This file contains all reviewer reports in order by version, followed by all author rebuttals in order by version.

Version 0:

Reviewer comments:

Reviewer #2

(Remarks to the Author)
The authors use their established system to study epithelial phagocytosis in the zebrafish embryonic epithelium (the enveloping layer, EVL). In earlier work they showed that the EVL can efficiently scavenger experimentally induced apoptotic cells, a common function of epithelia in many species. Cell death is induced by mosaically expressing the apoptotic protein Bax in embryonic deep cells during blastula stages. The authors examine the role of E-cadherin in the clearance of apoptotic cells and propose that E-cadherin is required to limit Myosin II activity at phagocytic contact sites. This function of E-cadherin appears to be mediated by p120-catenin and is independent of E-cadherin trans-binding. The authors propose that Ecad functions as a mechano-regulator of phagocytosis in the epithelium. The data is of high quality, and the authors have substantially revised the manuscript from an earlier version, including providing additional images and movies which are very helpful.

Remaining Points
The data demonstrating that E-cadherin and p120 knock-down results in increased basal myosin is convincing, as is the rescue by myosin perturbation. However, the fact that myosin perturbation has no effect on wild type embryos seems to call into question the importance of myosin for phagocytosis in normal circumstances. In my view, the control data for Figure 5 E&F should be shown in Figure 5 and not in the supplement. While additional experiments may be beyond the scope of this work, it seems worth addressing this point in the discussion. In addition, is basal actin also increased in E-cadherin morphants and could this be shown?

Just a note that the function of E-cadherin in phagocytosis is intriguing considering work from Nicole King's lab at Stanford on choanoflagellates. The unicellular choanoflagellates contain distantly related cadherin-like proteins that are used for engulfment of bacterial particles. In this sense, the findings here could be related to this ancient and potentially ancestral function of cadherins.

Reviewer #3

(Remarks to the Author)

The current revised manuscript presents an interesting new link between E-cadherin/catenin complex formation at the basal side of epithelia and apoptotic cell clearance in zebrafish embryos. I appreciate that the authors have gone through extensive revision work to improve the quality of the manuscript. They have now delivered more supporting evidence that the E-cadherin/catenin complex is responsive to phagocyte target contact, controls myosin 2 dynamics via p120 and regulates phagocytic uptake of apoptotic targets. The authors also claim that E-cad/catenin facilitates mechanical force transmission via the physical link of E-cad to the actin cytoskeleton promoting actin polymerisation. The experimental procedures seem technically sound and state-of-the art. The presented images from extensive live cell imaging experiments

are generally clear and compelling and the data analysis and statistical tests seem overall solid. I believe that the work is of high significance in the field and beyond.

However, I have several remaining concerns/comments about some of the experimental data and the conclusions drawn from the presented evidence.

• Epithelial arms and apoptotic target movements: How do the authors know that all the movements of the apoptotic targets are derived from epithelial arms? In many movies, the apoptotic bodies seem to be motile and self-propelled. Are all moving bodies associated with epithelial arms? For example, in Fig 4D and Suppl Fig 5E-G, the authors claim that formation of epithelial arms are fully functional but this is not clear from the target dispersal data alone.
Related, in Supplementary Fig. 2G-L, an alternative explanation for restricted spatial dispersion would be that if apoptotic cell speed is reduced (independent of arm protrusions), deep cells undergoing apoptosis might not reach the EVL for phagocytosis.

• Localisation of a/b-catenin to the basal epithelial surface: From the orthogonal projections such as in Fig 4A or Suppl Fig 5D, it is hard to clearly recognise if the signal is originated from the basal vs lateral side of the host cell vs a signal from the apoptotic target. To avoid misinterpretation, can the authors clearly point out in such figures where apical/lateral/basal of the host cell is located?
Further, is recruitment of a/b-catenin dependent on E-cadherin? For example, are a/b-catenin recruited to the apoptotic targets in E-cad MO embryos?

• E-cad/catenin regulating different actin force transmission pathways: Although plausible, this claim is currently weakly supported by the presented data and speculative. The proposed model in Fig. 4E is attractive and could potentially explain different mechanisms that regulate forces involved in engulfment of apoptotic fragments or growing of epithelial arms. However, although the authors have identified key players, I feel that the functional interactions between these and the pathways linking them to force transmission and actin polymerisation in the two proposed cases, are speculative as of now and not fully conclusive from the presented data. For example, observing actin dynamics under different scenarios would be helpful here. What is the role of E-cad in this process and how is it linked to a-catenin function? If E-cad generates friction at the surface which would counteract F-actin-mediated cup growth, I would expect that a-catenin removal would facilitate pushing forces and cup progression. How is frictional coupling between the actin cytoskeleton and the apoptotic target achieved without the need for E-cadherin-mediated cell-cell adhesion? I appreciate that dissecting the underlying mechanisms of these different pathways might take more time and may be beyond the scope of this manuscript. Sketching a model is useful to demonstrate potential mechanisms, but it should be treated as speculative for now rather than a conclusion of the findings.

• Role of myosin 2: The authors show that on one side, myosin 2 is recruited to the phagosome for cup progression and closure which suggests a functional role in this process. On the other side, inhibition of myosin 2 (blebbistatin, p120, E-cad) which would also affect cup elongation and closure, seems to facilitate phagocytosis. I am puzzled by the contradictory roles of myosin 2 in this process. How can recruitment/activation and downregulation of myosin 2 both have positive functions for phagocytosis?

• E-cadherin and cell cortex mechanics: The authors state that the change in tissue architecture depends on the contact with apoptotic cells in E-cadherin deficient epithelia (Suppl Fig. 6A,B). To show that this is E-cadherin dependent would require the same analysis in wt embryos. Does apoptotic cell interaction in wt lead to apical constriction as well?

• Role of a-cat: It is rather surprising to me that a-cat depletion does not affect myosin 2 enrichment, given that a-cat links E-cadherin to the actomyosin cortex and often controls actomyosin dynamics. If a-catenin's main function is to regulate action polymerisation, how would that work? A-catenin can regulate actin polymerisation by inhibiting Arp-2/3 which promotes actin branching, but this would likely be not productive here for cup progression, as the authors propose a bundled pushing network parallel to the junctions.

Downregulation of a-cat in many cases also affects E-cad stabilisation at the membrane. Did the author's check that E-cadherin is still localised to the basal epithelial surface under a-catenin depletion? As outcomes for E-cad and a-cat depletions regarding apoptotic clearance are different that would be important to check.

Minor comments:

• DECMA antibody experiments: The authors state that the blocking AB specifically targets basal E-cad without affecting lateral junctions (which they show in R21). DECMA has been shown to be very potent in breaking junctional E-cadherin mediated cell-cell adhesions in confluent monolayers. How do the authors explain the specific effect on basal vs lateral cadherin in the embryo? This seems quite surprising, also given the same outcomes of DECMA and E-cad MO experiments.

• The authors compare E-cad/catenin complex recruitment to the basal epithelial surface with nascent cell-cell contacts (discussion). However, recruitment at the latter is normally triggered by E-cadherin-mediated cell-cell adhesion. How does the complex get activated at the basal epithelial surface when trans-adhesion is absent/dispensable?

• The authors added a summary figure (Fig. 6c) which is useful. Given the discovery of multiple key players (including myosin 2) it would be helpful to add the relationships and dependencies between the proteins to the figure to highlight downstream/upstream regulators. Speculative pathways could be marked with a question mark.

• Suppl Fig. 7A. It is surprising that the authors find a significant difference given the strong overlap of the data points and the nearly identical mean cell area. I assume that this is related to the fact that the sample size is very unequal (2 fold) which reduces statistical power and affects results.

Version 1:

Reviewer comments:

Reviewer #2

(Remarks to the Author)
The authors propose that Ecad functions as a mechano-regulator of induced phagocytosis in the embryonic epithelium. The data is of high quality, and the authors have addressed my remaining concerns.

Reviewer #3

(Remarks to the Author)
The authors addressed all my questions sufficiently and I am happy with the revised version. I believe that this improved version of the manuscript warrants publication in Nature Communications and the presented outcomes will be of high importance in the field.

Open Access This Peer Review File is licensed under a Creative Commons Attribution 4.0 International License, which permits use, sharing, adaptation, distribution and reproduction in any medium or format, as long as you give appropriate credit to the original author(s) and the source, provide a link to the Creative Commons license, and indicate if changes were made.
In cases where reviewers are anonymous, credit should be given to 'Anonymous Referee' and the source.
The images or other third party material in this Peer Review File are included in the article's Creative Commons license, unless indicated otherwise in a credit line to the material. If material is not included in the article's Creative Commons license and your intended use is not permitted by statutory regulation or exceeds the permitted use, you will need to obtain permission directly from the copyright holder.
To view a copy of this license, visit https://creativecommons.org/licenses/by/4.0/

REPLY TO REVIEWERS

We sincerely thank the reviewers for their careful reading of our manuscript and for their constructive and insightful comments. Their feedback has been very helpful in strengthening the manuscript and improving the clarity of our work. All comments have been carefully considered, and we provide detailed point-by-point responses below.

Reviewer #2 (Remarks to the Author):

The authors use their established system to study epithelial phagocytosis in the zebrafish embryonic epithelium (the enveloping layer, EVL). In earlier work they showed that the EVL can efficiently scavenger experimentally induced apoptotic cells, a common function of epithelia in many species. Cell death is induced by mosaically expressing the apoptotic protein Bax in embryonic deep cells during blastula stages. The authors examine the role of E-cadherin in the clearance of apoptotic cells and propose that E-cadherin is required to limit Myosin II activity at phagocytic contact sites. This function of E-cadherin appears to be mediated by p120-catenin and is independent of E-cadherin trans-binding. The authors propose that Ecad functions as a mechano-regulator of phagocytosis in the epithelium. The data is of high quality, and the authors have substantially revised the manuscript from an earlier version, including providing additional images and movies which are very helpful.

We thank the reviewer for the positive evaluation of our work and their appreciation for the thorough work that has been performed during the revision process. Thanks to the reviewer's comments, we have now further revised the presentation and discussion of our results. Below we provide a detailed point-by-point response to address each comment.

Remaining Points

The data demonstrating that E-cadherin and p120 knock-down results in increased basal myosin is convincing, as is the rescue by myosin perturbation. However, the fact that myosin perturbation has no effect on wild type embryos seems to call into question the importance of myosin for phagocytosis in normal circumstances. In my view, the control data for Figure 5 E&F should be shown in Figure 5 and not in the supplement. While additional experiments may be beyond the scope of this work, it seems worth addressing this point in the discussion.

We thank the reviewer for appreciating our new results supporting the mechanistic link between E-cadherin, p120-catenin and Myosin II. We agree that the results obtained in control embryos are important to highlight. Accordingly, we have moved these results to the main figure (previously Supplementary Figure 7B,C; now Figure 5G,H) to better emphasize them. We found that the overall phagocytic efficiency is not affected by Myosin II downregulation following Blebbistatin treatment. This result is consistent with previous reports showing that Myosin II is dispensable for complement- and FcR-dependent phagocytosis (Jaumouille et al 2019 and Vorselen et al 2021), suggesting that it is also not essential for PS-dependent phagocytosis. Interestingly, further analysis of phagosome sizes showed that Blebbistatin treatment affects the size of uptaken phagosomes, which is increased both in control and E-cadherin deficient embryos (Fig. R1). These results suggest that, even if not essential, Myosin II is involved in regulating the uptake of small versus large particles and thus also the regulation of phagosome size. How such regulation is achieved is beyond the scope of this study and is an interesting direction for future work. To clarify the role of Myosin II in epithelial efferocytosis, we have further amended the discussion section (lines 536-539).

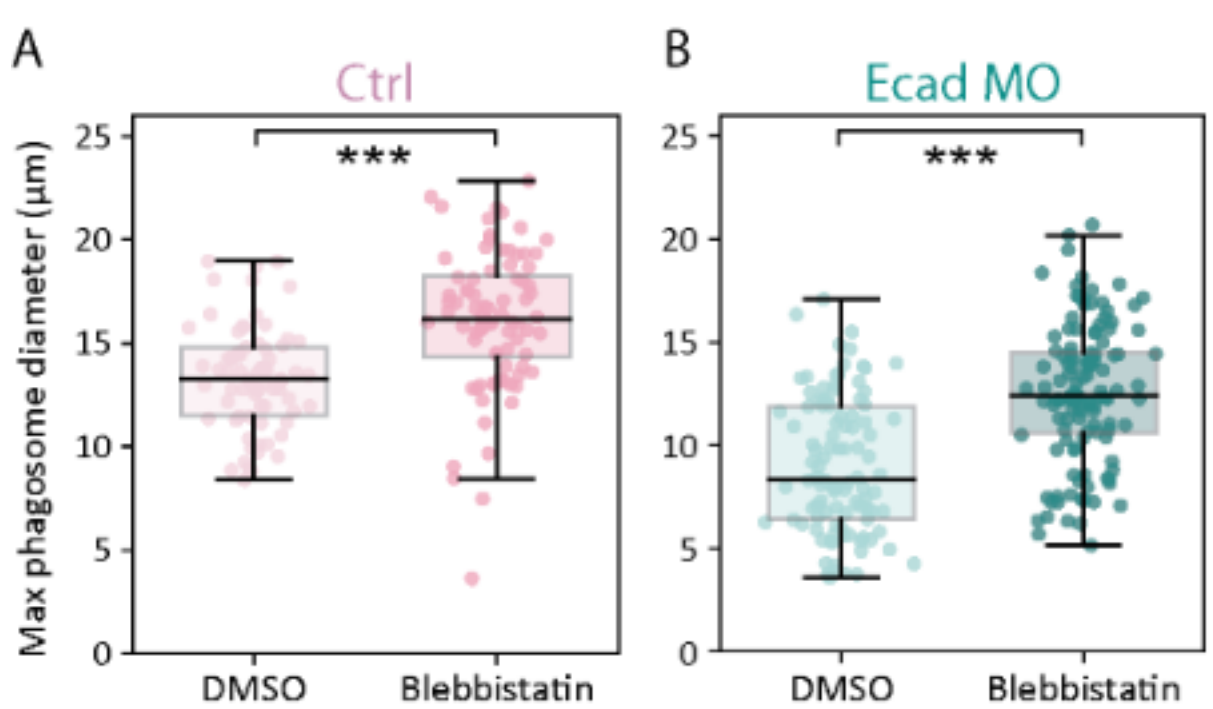

Figure R1. Myosin II influences phagosome sizes. A) *Diameter of the five largest phagosomes cleared by the epithelium in control embryos treated with DMSO (n =70 phagosomes from 14 embryos) or treated with Blebbistatin (n = 84 phagosomes from 20 embryos). Unpaired t-test, p = 0.003073.* ***B)*** *Diameter of the five largest phagosomes cleared by the epithelium in Ecad MO embryos treated with DMSO (n = 105 phagosomes from 21 embryos) or treated with Blebbistatin (n = 125 phagosomes from 25 embryos); Mann-Whitney test,* $p < 10^{-4}$.

In addition, is basal actin also increased in E-cadherin morphants and could this be shown?

We thank the reviewer for bringing up this point. We now provide additional data which show that in E-deficient epithelial cells, actin is remodeled at the phagocyte-target contact site and protrusions are initiated, supporting that PS-recognition is functional even in E-cadherin deficient epithelial cells (Fig. R2, Supplementary Figure 2G,H,Supplementary Movie 8). However, these initial protrusions do not develop into functional phagocytic cups, as shown by their altered morphology, and thus target engulfment fails in E-cadherin deficient versus control epithelial cells. While actin remodeling is initiated in both control and E-cadherin deficient

epithelial cells, our results confirm that an excessive Myosin II enrichment at the phagocyte-target contact sites hampers proper protrusion formation in E-cadherin or p120-catenin deficient epithelial cells (Figure 5C,D, Supplementary Figure 6E,8F). We thus conclude that E-cadherin is not required for actin remodeling *per se* but for keeping Myosin II activity in check through the recruitment of the p120-catenin/E-cadherin complex, which ensures the correct phagocytic clearance of apoptotic cells.

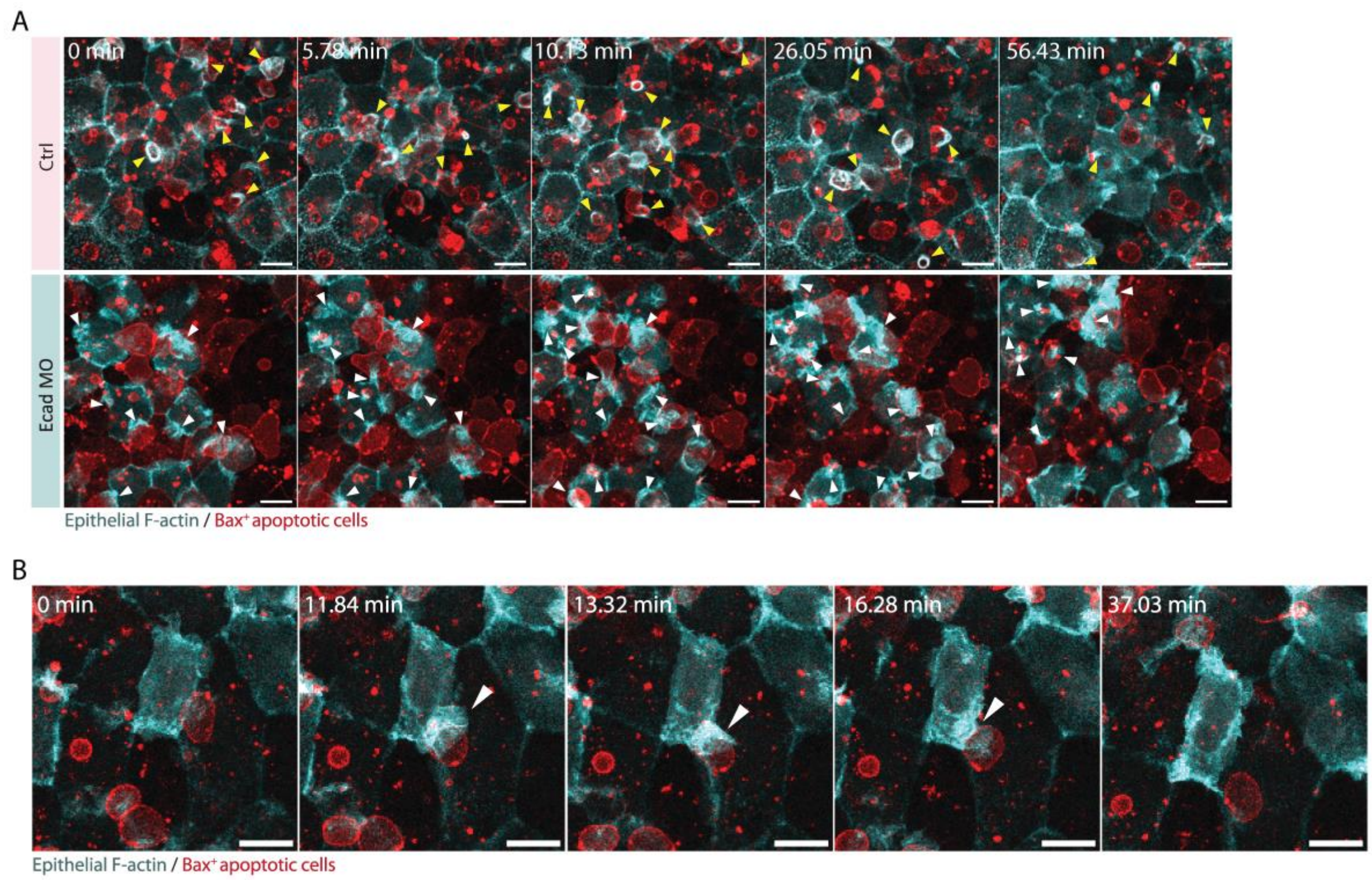

Figure R2. Reduction of E-cadherin affects epithelial actin dynamics and impairs actin based protrusion morphology in vivo. ***(A)*** *Time lapse dual color max z-projections images (Δz=4 µm; overlay) showing epithelial tissue F-actin dynamics in control (top) and E-cadherin MO injected (bottom) embryos expressing Lifefact-GFP (cyan) specifically in the epithelial layer under the krt18 promoter. Yellow arrowheads highlight successful phagocytic cups in control embryos, while white arrowheads point towards stalled phagocytic cups in E-cadherin MO embryos.* ***(B)*** *Time lapse dual color max z-projections images (Δz=6 µm; overlay) of a single stalled phagocytic cup (white arrowhead) around an apoptotic cell (red) in an E-cadherin morphant embryo expressing Lifefact-GFP (cyan) specifically in the epithelial layer under the krt18 promoter. Embryos were obtained from the Tg(Krt18:Gal4FF/UAS:Lifeact-GFP) line (A, B). Scale bars: 20 µm (A,B).*

Just a note that the function of E-cadherin in phagocytosis is intriguing considering work from Nicole King's lab at Stanford on choanoflagellates. The unicellular choanoflagellates contain

distantly related cadherin-like proteins that are used for engulfment of bacterial particles. In this sense, the findings here could be related to this ancient and potentially ancestral function of cadherins.

We thank the reviewer for referring to the interesting research of Nicole King's lab. King's team has described that Choanoflagellates, the closest unicellular relatives of metazoans, contain many cadherins that are not directly involved in cell-to-cell adhesion or multicellularity (Nichols et al., 2012; King et al., 2008; Abedin and King, 2008). Their research shows that cadherins and other adhesive molecules predate animal multicellularity origin, and consequently they must have had other ancestral functions. Particularly, Cadherins from non-colonial *Monosiga brevicollis* are localized at actin rich microvilli of the feeding collar, suggesting they may participate in the phagocytosis of bacterial prey (Abedin and King, 2008). The capacity of cadherins to promote bacterial uptake is supported by the exploitation of E-cadherin by pathogenic bacteria during host cell invasion, which we already discuss in our manuscript.

As suggested by the reviewer, we have now included a reference to Nicole King's research on the potential ancestral role of cadherins during phagocytosis in the discussion (lines 571-574).

Reviewer #3 (Remarks to the Author):

The current revised manuscript presents an interesting new link between E-cadherin/catenin complex formation at the basal side of epithelia and apoptotic cell clearance in zebrafish embryos. I appreciate that the authors have gone through extensive revision work to improve the quality of the manuscript. They have now delivered more supporting evidence that the E-cadherin/catenin complex is responsive to phagocyte target contact, controls myosin 2 dynamics via p120 and regulates phagocytic uptake of apoptotic targets. The authors also claim that E-cad/catenin facilitates mechanical force transmission via the physical link of E-cad to the actin cytoskeleton promoting actin polymerisation. The experimental procedures seem technically sound and state-of-the art. The presented images from extensive live cell imaging experiments are generally clear and compelling and the data analysis and statistical tests seem overall solid. I believe that the work is of high significance in the field and beyond.

However, I have several remaining concerns/comments about some of the experimental data and the conclusions drawn from the presented evidence.
We thank the reviewer for the positive evaluation of our work and for acknowledging the substantial effort undertaken during the revision work. Following the reviewer's comments, we have now further revised the presentation and discussion of our results and added more data to

clarify the mechanistic insights of our working model. Below we provide a detailed point-by-point response to address each comment.

• Epithelial arms and apoptotic target movements: How do the authors know that all the movements of the apoptotic targets are derived from epithelial arms? In many movies, the apoptotic bodies seem to be motile and self-propelled. Are all moving bodies associated with epithelial arms? For example, in Fig 4D and Suppl Fig 5E-G, the authors claim that formation of epithelial arms are fully functional but this is not clear from the target dispersal data alone. Related, in Supplementary Fig. 2G-L, an alternative explanation for restricted spatial dispersion would be that if apoptotic cell speed is reduced (independent of arm protrusions), deep cells undergoing apoptosis might not reach the EVL for phagocytosis.

As correctly pointed out by the reviewer, apoptotic targets are very mobile in the embryo interior. In previous work, in which epithelial arms were described for the first time (Hoijman et al, 2021), we performed multiple orthogonal experiments which showed that 1) apoptotic targets do not have an autonomous motility through specifically interfering with the actomyosin machinery *in vivo* and further assessing apoptotic cell motility *in vitro* (Hoijman et al, 2021 , Extended Data Fig. 7c, d) and 2) our results showed that apoptotic motility is not a cell autonomous process but is instead directly dependent on forces generated by epithelial pushing protrusions (Hoijman et al, 2021 Extended Data Fig. 9g, h). Both apoptotic target dispersal at the tissue level and apoptotic target speed for individual targets therefore require the activity of epithelial arm protrusions. Tracking of apoptotic targets and analyzing target speed thus represents the best proxy to quantify epithelial arm formation *in vivo*.

We further show here that specific interference with E-cadherin complex members has distinct effects on apoptotic target dispersal: in p120-catenin/E-cadherin deficient epithelial cells target dispersal is compromised, while a-catenin deficient or mutant variants maintain an intact target dispersal. In addition, E-cadherin downregulation exclusively in apoptotic cells did not affect apoptotic cell mobility (Supplementary Figure 4 A-D), further supporting that apoptotic cell mobility is directly dependent on epithelial protrusion activity. Moreover, synthetic targets (lipid aggregates) that do not have an intrinsic mobility, show a similar dispersal as apoptotic targets (Figure 3). Together, these results support that apoptotic target dispersal is not regulated by a cell intrinsic mechanism but is rather controlled through the protrusion formation in epithelial cells which is regulated by the E-cadherin/catenin complex *in vivo*. We have now revised the main text to clarify the role of epithelial arms in apoptotic cell mobility and the involvement of the E-cadherin/catenin complex (lines 129-134).

• Localisation of a/b-catenin to the basal epithelial surface: From the orthogonal projections such as in Fig 4A or Suppl Fig 5D, it is hard to clearly recognise if the signal is originated from the basal vs lateral side of the host cell vs a signal from the apoptotic target. To avoid

misinterpretation, can the authors clearly point out in such figures where apical/lateral/basal of the host cell is located?
Further, is recruitment of a/b-catenin dependent on E-cadherin? For example, are a/b-catenin recruited to the apoptotic targets in E-cad MO embryos?

We appreciate the constructive comment on the data presentation. As suggested by the reviewer, we have now indicated where the apical, lateral and basal sides of the epithelial phagocytic cell are located at the first image of the timelapse (Fig. R3, Figure 4A and Supplementary Figure 5C). Moreover, we would like to clarify that in our α/β-catenin localization experiments, we transplanted apoptotic cells from a donor embryo co-expressing Bax and a membrane marker (Lyn-tdTomato/Cerulean, without the expression of tagged catenins), into a host embryo expressing α-catenin-mCherry or EGFP-β-catenin. In this way, we ensured that we were visualizing the α/β-catenin signal exclusively from epithelial cells at the phagocytic contact sites. This is mentioned in the main text (lines 244-247) and we have now also added the information to the figure legends of Figure 4A and Supplementary Figure 5C.

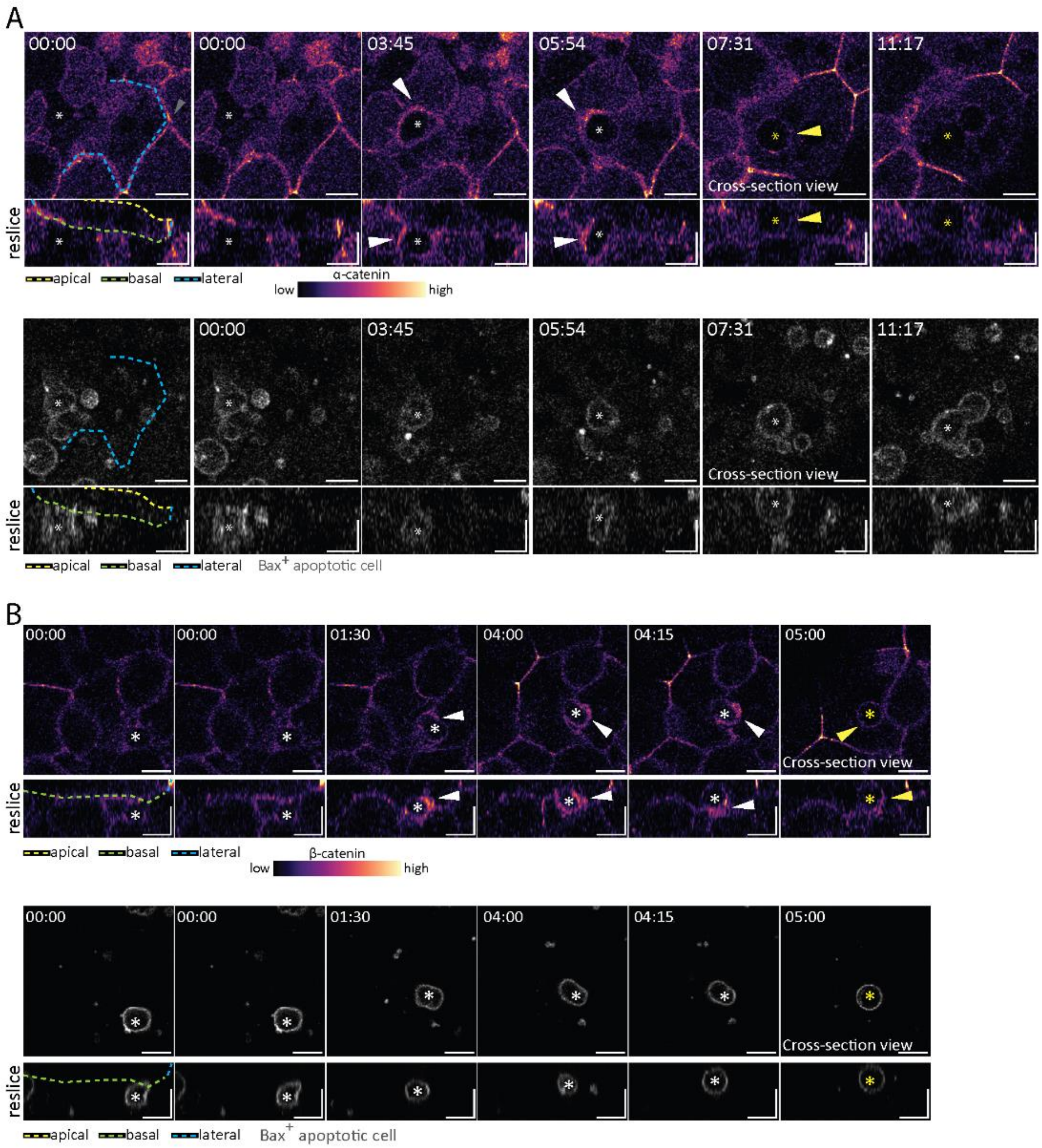

Figure R3. α and β-catenin localize at the phagocytic synapse during epithelial uptake of apoptotic cells. ***A)*** *Time lapse images showing the localization of α-catenin-mCherry (top pannels) during the uptake of a transplanted apoptotic cell target (Bax+ cells co-expressing Lyn-Cerulean, bottom panels) by epithelial cells in an embryo expressing α-catenin-mCherry. White arrowheads indicate α -catenin localization during the uptake of an apoptotic cell, while yellow arrows show the localization of α -catenin at the newly formed phagosome. Dashed lines outline the different surfaces of the phagocytic epithelial cell at time 0.* ***B)*** *Time lapse images showing the localization of β-catenin (white arrowheads, top panels) during the uptake of a transplanted apoptotic cell target (Bax+ cells co-expressing Lyn-tdTomato, bottom panels) by epithelial cells in an embryo expressing β-catenin-GFP. White arrowheads*

indicate β-catenin localization during the uptake of an apoptotic cell, while yellow arrows show the localization of β-catenin at the newly formed phagosome. Dashed lines outline the different surfaces of the phagocytic epithelial cell at time 0. Scale bars x: 10 µm, z: 10 µm (A, B).

Lastly, we aimed to understand if the recruitment of α-catenin is dependent on E-cadherin. For this purpose, we measured the localization of α-catenin-mCherry in E-cadherin deficient embryos transplanted with apoptotic cells devoid of α-catenin-mCherry signal. As expected for a reduction of junctional E-cadherin levels, the junctional localization of α-catenin-mCherry was also reduced compared to control embryos, associated with a significantly increased cytosolic localization (Fig. R4A). At the target interface, E-cadherin deficient epithelial cells did not show a clear accumulation of α-catenin-mCherry signal (Fig. R4B, Supplementary Figure 5B); supporting the basal α-catenin recruitment at the phagocytic cup is dependent on E-cadherin. A technical limitation of our assay is that the increased cytosolic levels of α-catenin-mCherry signal in E-cadherin deficient epithelial cells might hinder the visualization of a reduced local enrichment of α-catenin that can occur considering the residual pool of E-cadherin in E-cadherin morphant embryos. While E-cadherin deficient embryos have a strong reduction in phagocytic activity (Figure 1H), there are few occasions where epithelial cells engulf apoptotic cells, particularly those targets of smaller size (Supplementary Figure 2F). We provide an example of such a rare event in E-cadherin deficient embryos, for which we observed a mild α-catenin-mCherry accumulation at the phagocytic cup (Fig. R4C). This observation supports that α-catenin recruitment takes place exclusively during successful uptake events, which are rare events restricted to small apoptotic targe sizes in E-cadherin deficient epithelial cells.

Our conclusion that α-catenin recruitment at the phagocytic cup is critical for successful phagocytic uptake is further supported by our following data: First, we show that the actin-binding site of α-catenin is essential for phagocytosis even under normal E-cadherin levels (Figure 4F), supporting that the physical linkage of the E-cadherin/catenin complex to actin is essential for phagocytosis to occur. Notably, the clearance defect for reduced E-cadherin levels can be partially rescued by Myosin II inhibition (Figure 5E, F), which directly supports that a residual physical α-catenin linkage to actin is present even at reduced E-cadherin expression levels. Additionally, we show that α-catenin is not involved in the regulation of actin polymerization (epithelial arm protrusions activity is normal) or in the regulation of basal epithelial cell cortex mechanics at the phagocytic synapse (no alterations in Myosin II levels upon α-catenin depletion). See more details about these points in the reply below. Together, these data support that 1) α-catenin recruitment to the target site is dependent on E-cadherin, and 2) E-cadherin/α-catenin complex binding to F-actin is critical for phagocytosis to occur.

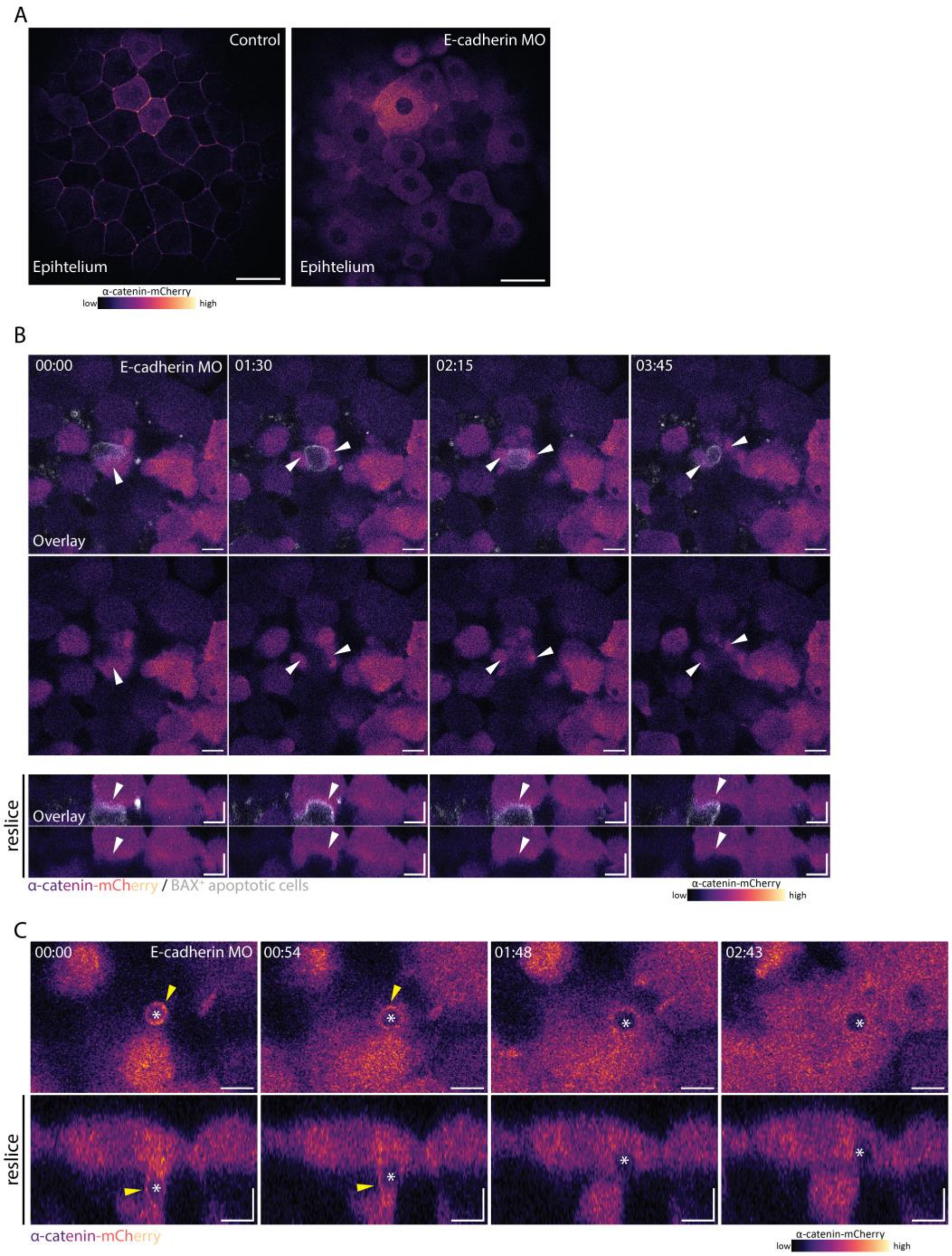

Figure R4. α-catenin does not enrich at the phagocyte-target interface in E-cadherin downregulated epithelial cells. *(**A**) Representative dual colour fluorescence images showing the localization of α-catenin-mCherry in control (left) and Ecad MO injected embryos (right) at sphere (4 hpf) stage. (**B**) Fluorescence time lapse images showing the localization of α-catenin-mCherry in epithelial cells in contact with transplanted apoptotic cells (grey, Bax+ cells) in Ecad MO injected embryos at gastrula stage (5 hpf). White arrowheads indicate the absence of local enrichment of α-catenin-mCherry at the contact side with*

*targets. (**C**) Fluorescence time lapse images showing the localization of α-catenin-mCherry in epithelial cells during a successful phagocytic uptake of an apoptotic target (asterisk) in Ecad MO injected embryos at gastrula stage (5 hpf). Yellow arrowheads indicate α-catenin-mCherry localization at the phagocytic cup. Scale 20 µm (A), 10 µm (B, C).*

• E-cad/catenin regulating different actin force transmission pathways: Although plausible, this claim is currently weakly supported by the presented data and speculative. The proposed model in Fig. 4E is attractive and could potentially explain different mechanisms that regulate forces involved in engulfment of apoptotic fragments or growing of epithelial arms. However, although the authors have identified key players, I feel that the functional interactions between these and the pathways linking them to force transmission and actin polymerisation in the two proposed cases, are speculative as of now and not fully conclusive from the presented data. For example, observing actin dynamics under different scenarios would be helpful here. What is the role of E-cad in this process and how is it linked to a-catenin function? If E-cad generates friction at the surface which would counteract F-actin-mediated cup growth, I would expect that a-catenin removal would facilitate pushing forces and cup progression. How is frictional coupling between the actin cytoskeleton and the apoptotic target achieved without the need for E-cadherin-mediated cell-cell adhesion? I appreciate that dissecting the underlying mechanisms of these different pathways might take more time and may be beyond the scope of this manuscript. Sketching a model is useful to demonstrate potential mechanisms, but it should be treated as speculative for now rather than a conclusion of the findings.

We appreciate this comment and we would like to further clarify the role of α-catenin in epithelial phagocytosis and our working model. Phagocytosis of large particles, such as apoptotic cells, requires a large deformation of the phagocyte surface to engulf the target. This is achieved by 1) actin polymerization, that pushes the leading edge of the protrusion forward, and 2) adhesive anchors, that connect the target with the cytoskeleton of the phagocyte and consequently, guide the protrusion around the target's surface (Jaumouillé & Waterman, 2020). Similar to focal adhesions, the physical coupling between the anchors at the membrane and the cytoskeleton prevents actin retrograde flow. In addition, the transmission of forces generated by actin polymerization to the substrate facilitates a forward extension of the phagocytic cup, which is necessary for target engulfment. In Complement- and FcR mediated phagocytosis (Jaumouille et al 2019, Settle et al, 2024), Integrin β2 can form a complex that connects the phagocytic target with the phagocyte cytoskeleton. Notably, force transmission in efferocytosis is still largely unresolved.

In our work, we propose that the E-cadherin/catenin complex plays a similar role to Integrin β2 during the phagocytic clearance of apoptotic cells by epithelia. The role of α-catenin as the physical linker between F-actin and E-cadherin at the phagocytic cup is further supported by several evidence. First, our results support that E-cadherin is localized at the phagocytic cup (Figure 1E), associated with the consecutive recruitment of β- and α-catenin, the latter being able

to directly bind to F-actin (Figure 4A, Supplementary Figure 5C). Second, interference with the α-catenin binding site to actin through expression of a mutant α-catenin construct fully compromises phagocytic clearance, supporting the critical role of a physical coupling to actin through the E-cadherin/ α-catenin complex (Figure 4F). Third, the formation of epithelial arm protrusions – which, instead of wrapping around the target, push apoptotic targets away - is fully functional in epithelial cells depleted of α-catenin or expression the α-catenin binding mutant (Figure 4D, Supplementary Figure 5H-J; Supplementary Movie 18, Supplementary Movie 19). This can be understood since the pushing of targets requires normal forces that can act perpendicular to the target surface. In contrast, the formation of a membrane protrusion along the target during phagocytic cup formation requires lateral/tangential forces that counteract the actin polymerization forces to push the membrane protrusion forward.

Notably, PS-receptors generally lack actin binding motifs, making it unclear how forces from actin polymerization are coupled to protrusion formation and phagocytosis. Our results support that α-catenin establishes the relevant missing physical link to the actin cytoskeleton, which can explain how forces from actin polymerization are mechanically translated into the forward advancement of a phagocytic cup.

We would like to clarify, that we have not yet identified the E-cadherin binding partner at the apoptotic cell surface. We observed that E-cadherin is dynamically localized to the phagocytic cup, associated with the rapid recruitment of α-, β- and p120-catenin. This strongly supports its specific engagement and functional role at the site of PS-receptor engagement and PS ligand recognition. Based on experiments with synthetic targets that lack E-cadherin, we can exclude the trans-binding of E-cadherins is necessary for target engulfment. We speculate that E-cadherin might be able to directly bind Phosphatidylserine through its EC1 domain, or act as a PS co-receptor at the epithelial surface. Identifying these binding targets of E-cadherin are an interesting future aspect to address. Despite currently lacking mechanistic insight into the binding of the extracellular E-cadherin domain at the phagocytic synapse, we believe that our work here provides important novel insight by dissecting the role of the E-cadherin complex and its downstream effectors in apoptotic cell clearance, which unraveled novel functions of E-cadherin in this process: 1) we identify a mechano-signaling function of the E-cadherin/catenin complex to regulate Myosin II activity at the phagocytic synapse and 2) we find that the E-cadherin/catenin complex establishes a physical link to the actin cytoskeleton for mechanical force transmission, which significantly advances our limited understanding of epithelial efferocytosis and the underlying mechanisms.

Following the Reviewer's comment, we have rewritten the manuscript to better clarify the role of α-catenin during phagocytosis and target dispersal. We now also refer to α-catenin as the physical linker between the apoptotic target and the phagocyte cytoskeleton, and we avoid terms like frictional coupling to emphasize the role of E-cadherin/catenin as a physical linker to the actin cytoskeleton.

• Role of myosin 2: The authors show that on one side, myosin 2 is recruited to the phagosome for cup progression and closure which suggests a functional role in this process. On the other side, inhibition of myosin 2 (blebbistatin, p120, E-cad) which would also affect cup elongation and closure, seems to facilitate phagocytosis. I am puzzled by the contradictory roles of myosin 2 in this process. How can recruitment/activation and downregulation of myosin 2 both have positive functions for phagocytosis?

Our results suggest that 1) in our system, Myosin II is transiently recruited to the phagocytic cup during cup elongation (in line with Krendel & Gauthier, 2022; Swanson et al., 1999; Vorselen et al., 2021) and quickly removed from the phagocyte-target interface once the target is uptaken; 2) Myosin II recruitment is not essential for cup formation, since inhibition of Myosin II does not impair phagocytic uptake (similarly to what has been shown in complement- and antibody-dependent phagocytosis, Jaumouille et al 2019 and Vorselen et al 2021); and 3) overactivation of Myosin II, observed in E-cadherin or p120-catenin deficient embryos, is negatively affecting phagocytic cup progression. We thus conclude that Myosin II recruitment to the target interface requires a tight spatio-temporal control to allow for phagocytic uptake and an extended Myosin II recruitment/activity is detrimental to phagocytic target uptake. We show that the regulation of Myosin II is achieved through the presence of the E-cadherin/catenin complex at the phagocytic synapse, and specifically through the recruitment of p120-catenin. This elucidates a novel regulatory pathway of the E-cadherin/catenin complex in controlling epithelial cell cortex mechanics during the phagocytosis of apoptotic cells.

• E-cadherin and cell cortex mechanics: The authors state that the change in tissue architecture depends on the contact with apoptotic cells in E-cadherin deficient epithelia (Suppl Fig. 6A,B). To show that this is E-cadherin dependent would require the same analysis in wt embryos. Does apoptotic cell interaction in wt lead to apical constriction as well?

We agree with the reviewer that analyzing epithelial tissue architecture during apoptotic cell phagocytosis in WT embryos is important. Indeed, in our previous manuscript we describe it in detail during the first section of the results (lines 96-120). We observed that 1) apical epithelial cell area was maintained during ingestion of apoptotic targets (Figure 1C, D), and 2) epithelial cells in contact with apoptotic cells or live cells had comparable apical cell area (Supplementary Figure 1A). These data suggest that basal and apical domains of phagocytic epithelial cells are mechanically decoupled in WT embryos. By contrast, E-cadherin deficient epithelial cells show pronounced shape changes upon interactions with apoptotic targets (Supplementary Figure 6 A,B). These results support that E-cadherin is required for the phagocytic clearance function in epithelial cells and for maintaining epithelial architecture during the phagocytic clearance process.

• Role of a-cat: It is rather surprising to me that a-cat depletion does not affect myosin 2

enrichment, given that a-cat links E-cadherin to the actomyosin cortex and often controls actomyosin dynamics. If a-catenin's main function is to regulate action polymerisation, how would that work? A-catenin can regulate actin polymerisation by inhibiting Arp-2/3 which promotes actin branching, but this would likely be not productive here for cup progression, as the authors propose a bundled pushing network parallel to the junctions.

We agree with the reviewer that α-catenin has been shown to regulate the actomyosin cortex in other contexts, including the inhibition of Arp2/3 via competition for binding sites in F-actin bundles (Drees et al., 2005) or via its interactor Vinculin (James et al, 2024 ; Favarin et al., 2026).

In our previous work, we showed both phagocytic cup and epithelial arm formation require branched F-actin polymerization dependent on Arp2/3 nucleator and Rac1 (Hoijman et al, 2021). Inhibition of Arp2/3 via CK-666 or Rac1 via the overexpression of a dominant negative form of the protein abrogated the formation of both phagocytic cups and epithelial arms (Hoijman et al, 2021, Fig 3c-e,i-n and Extended Data Fig. 2h,i). By contrast, α-catenin downregulation does not impair epithelial arm formation (Figure 4D, Supplementary Figure 5H-J; Supplementary Movie 18, Supplementary Movie 19). Thus, our data support that α-catenin does not regulate F-actin polymerization at the phagocyte-target interface through Rac1 or the Arp2/3 complex. In addition, our data support that α-catenin is further not required for the control of Myosin II activity and the clearance defect cannot be rescued by Blebbistatin treatment (Supplementary Figure 9A,B). Instead, we propose a novel role of α-catenin as a physical linker between the F-actin network and E-cadherin (see also more details in previous points).

Downregulation of a-cat in many cases also affects E-cad stabilisation at the membrane. Did the author's check that E-cadherin is still localised to the basal epithelial surface under a-catenin depletion? As outcomes for E-cad and a-cat depletions regarding apoptotic clearance are different that would be important to check.

We agree with the reviewer that interference with E-cadherin complex members can potentially affect E-cadherin localization. We have previously validated that p120-catenin deficient embryos maintain similar E-cadherin levels through Western Blotting and quantitative imaging of protein levels at cell-cell junctions (Supplementary Figure 8D,E). To answer the reviewer question, we have now performed additional quantitative imaging of endogenous E-cadherin protein levels at the junctional and basal side of epithelial cells in α-catenin depleted embryos using the *KI(mlanYFP)*^{xt17}*cdh1-YFP* line. Our data show that E-cadherin maintains its localization both at the junctional and basal side of epithelial cells in α-catenin depleted embryos (Fig. R5 A-C,

Supplementary Figure 5F, G). These data support that impairment of phagocytic efficiency in α-catenin depleted embryos is not due to a destabilization of E-cadherin at the membrane.

Instead of altering the E-cadherin pool, our results therefore support that α-catenin has a different role in regulating the phagocytic progress. We propose that α-catenin acts as a physical linker between E-cadherin and the actin cytoskeleton, given its direct binding capacity with both actin and E-cadherin. This hypothesis is directly supported by expressing an α-catenin mutant version that can bind to E-cadherin but lacks the Actin Binding Site (α-catenin-ΔABS).

We interpret our data on α-catenin in the following manner: Wrapping of a membrane protrusion around an apoptotic target requires the forward propulsion of the leading edge by actin-mediated pushing forces. However, PS receptors generally do not have a direct physical link to the actin cytoskeleton through their cytoplasmic domains, leaving a critical gap in our understanding of how force transmission is achieved in efferocytosis. Our findings establish a relevant missing link between PS-receptor signaling and the mechanical force coupling in non-professional epithelial phagocytes: to translate the forces of actin polymerization into an effective forward propulsion of the phagocytic leading edge during target engulfment, the actin cytoskeleton needs to be physically linked to the target surface. If this physical coupling fails, the forces generated by actin cannot push the leading edge membrane and the formation of phagocytic protrusions is impeded. Instead, actin polymerization results in a rearward flow of the actin, as an anchoring of the actin cytoskeleton is required to translate actin polymerization into the forward growth of the phagocytic cup. In line with this physical coupling, a high retrograde cortical flow occurs in epithelial 'arms' as we have previously shown (Hoijman et al. Nature 2021). Our model is further in line with work on complement mediated phagocytosis (Jamouille et al. NCB 2019), in which Integrins serve as critical physical bridge to the actin cytoskeleton. From our results, we therefore derive at the following conclusions: α-catenin binding to actin is essential for phagocytosis to occur, providing a new physical linker in PS-mediated phagocytosis by epithelial cells.

We have now described the effects of α-catenin downregulation on E-cadherin localization in the text (lines 270-272) and included the newly acquired data in Supplementary Figure 5F,G.

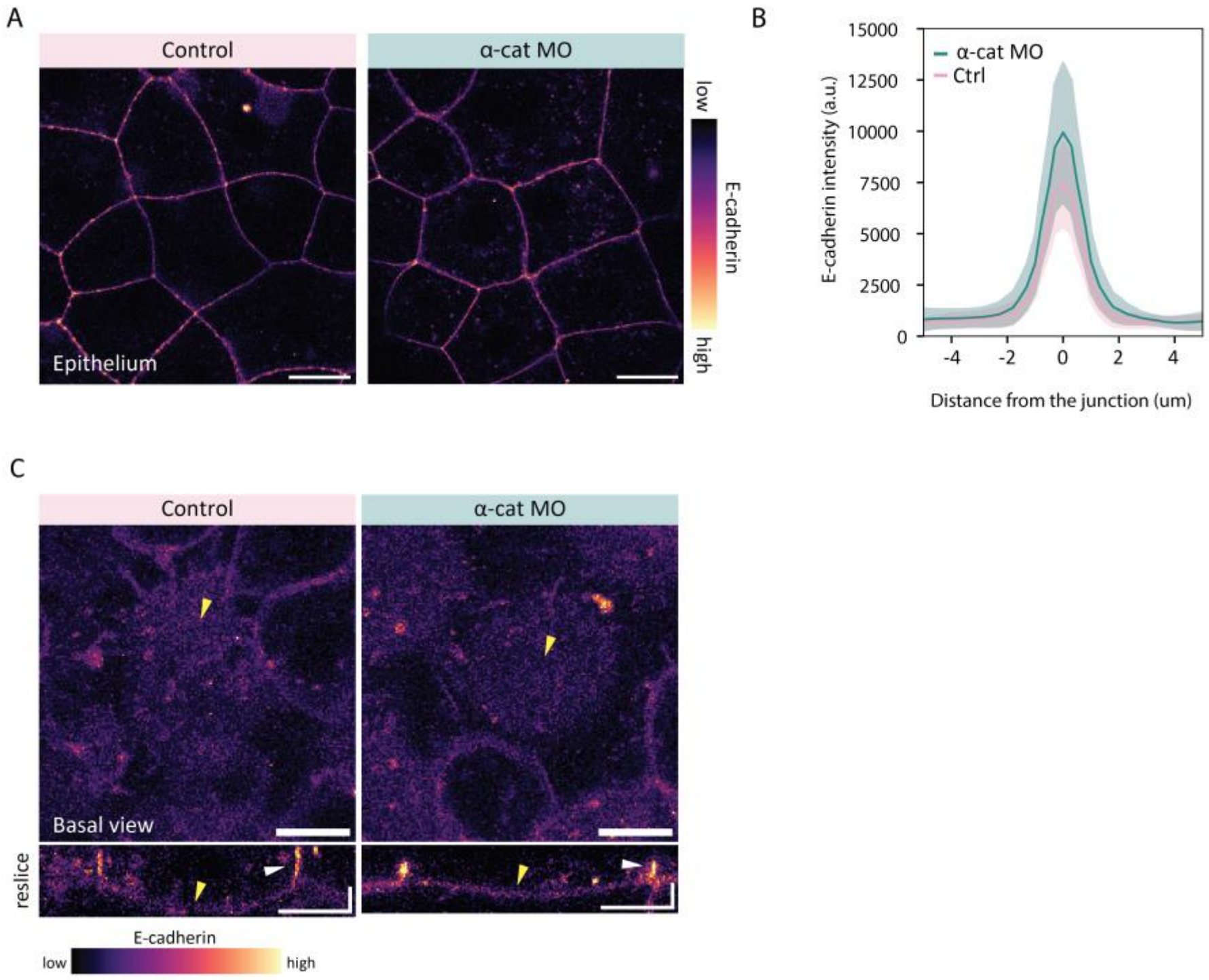

Figure R5. E-cadherin basolateral localization is maintained in α-catenin depleted embryos. A) *Representative images of epithelial cells at shield stage (6 hpf) in control and α-catenin MO (4 ng) injected KI(mlanYFP)*^{xt17}*cdh1-YFP embryos.* ***B)*** *Quantification of the E-cadherin signal intensity at epithelial cell junctions in control (pink, n = 55 junctions from 6 embryos, N = 2) and α-cat MO embryos (cyan; n = 35 junctions from 5 embryos, N = 2) at shield stage (6 hpf). Data lines and shaded areas represent the mean and SD.* ***C)*** *Basal view (top) and transversal view (bottom) showing E-cadherin localization in an epithelial cell in control (left) and α-catenin MO (right) embryos at shield stage (6 hpf). White arrowheads indicate the localization of E-cadherin at the cell-cell epithelial junctions; yellow arrowheads indicate the basal localization of E-cadherin. Embryos were obtained from the KI(mlanYFP)*^{xt17}*cdh1-YFP line. Scale bars: 20µm (A), x: 10 µm, z: 5 µm (C).*

Minor comments:

• DECMA antibody experiments: The authors state that the blocking AB specifically targets basal E-cad without affecting lateral junctions (which they show in R21). DECMA has been shown to be very potent in breaking junctional E-cadherin mediated cell-cell adhesions in confluent monolayers. How do the authors explain the specific effect on basal vs lateral cadherin in the embryo? This seems quite surprising, also given the same outcomes of DECMA and E-cad MO experiments.

The reviewer is correct that DECMA-1 does not penetrate the lateral epithelial cell-cell junctions in the studied timeframe (up to 2h after injections). This observation is supported by the data shown in R21, Supplementary Figure 3G, and the fact that epithelial integrity is not affected in

DECMA-1 injected embryos for the studied timeframe, as shown by the survival of the embryos. The difference to other systems could be caused by variable concentrations and/or the short incubation time in our assay (1-3 hours). For MDCK confluent monolayers, for example, Vestweber and Kemler 1985 showed that 100 µg/mL of DECMA-1 was impairing junctional E-cadherin mediated cell-cell adhesions after 5 h of incubation. In mESCs layers, DECMA-1 treatment resulted in the loss of cell-cell contact after 24 h of incubation (Segal & Ward, 2017). Moreover, differences in cell-cell contacts permeability between different epithelial tissues/cell lines could account for variable effects of DECMA-1 on the junctional pool of E-cadherin.

• The authors compare E-cad/catenin complex recruitment to the basal epithelial surface with nascent cell-cell contacts (discussion). However, recruitment at the latter is normally triggered by E-cadherin-mediated cell-cell adhesion. How does the complex get activated at the basal epithelial surface when trans-adhesion is absent/dispensable?

We agree with the reviewer that the absence of E-cadherin trans-interactions is one of the main differences between adherens junctions and the E-cadherin/catenin complex formed at the phagocytic cup. However, other mechanisms that have been described in the context of cell-cell contacts and beyond might take place at the phagocytic synapse. For example, cis-clustering of E-cadherin molecules is a key step during the formation of nascent junctions (Wu et al, 2015). Additionally, in non-junctional contexts like single cell migration (Grimaldi et al., 2020) or cytokinesis (Padmanabhan et al., 2017) ; Mondal et al., 2026), E-cadherin cis-interactions are sufficient to trigger the recruitment of catenins and its subsequent actomyosin regulation. Lastly, E-cadherin can bind many other proteins, forming heterotypic cis- and trans- interactions (Shafraz et al., 2020). We speculate that E-cadherin might act as a co-receptor and associated with epithelial PS receptor(s), and therefore, trigger the formation of the E-cadherin/catenin complex formation. Alternatively, the EC1 domain might directly interact with PS or other molecules at the apoptotic cell surface. Both the identification of the scavenger receptor(s) and other E-cadherin interactors at the phagocytic synapse are an interesting direction for future studies and is out of the scope of the current study.

We have now added part of this discussion into the manuscript (lines 556-566).

• The authors added a summary figure (Fig. 6c) which is useful. Given the discovery of multiple key players (including myosin 2) it would be helpful to add the relationships and dependencies between the proteins to the figure to highlight downstream/upstream regulators. Speculative pathways could be marked with a question mark.

Following the reviewers' suggestion, we have updated the summary figure (Figure 6C, Fig R6). We have now included other key players, like Myosin II and F-actin, and explicitly showed the relationship between them. Particularly, we include the inhibition of Myosin II by p120-catenin and the role of α/β-catenin connecting E-cadherin with the F-actin cytoskeleton during phagocytic cup formation. Additionally, we included the E-cadherin knock-down scenario, in which E-cadherin does not cluster at the phagocyte-target contact site and consequently, catenins are not recruited. Under this context, p120-catenin cannot inhibit Myosin II and α/β-catenin lose their connection to the cytoskeleton.

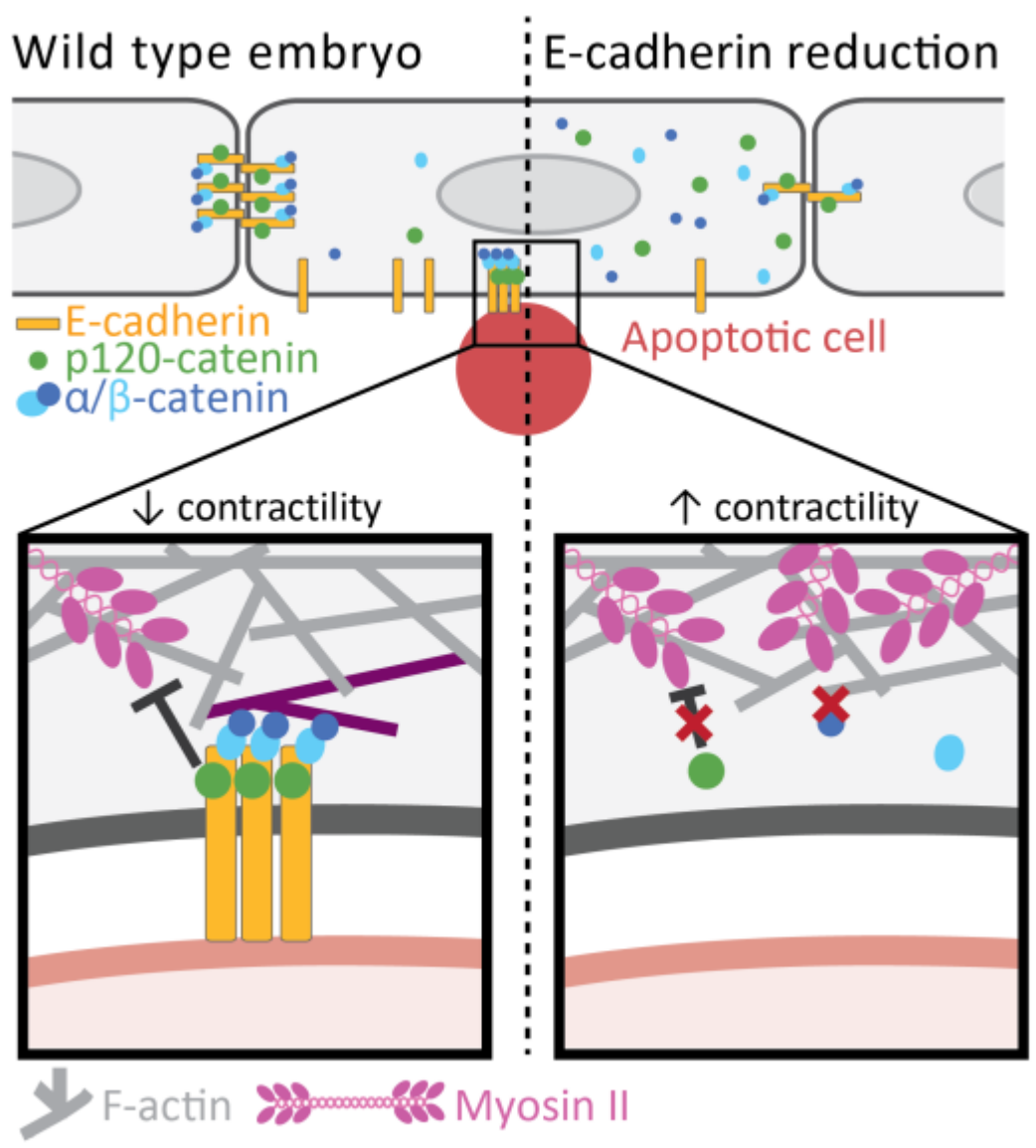

Fig R 6. E-cadherin/catenin complex formation during apoptotic cell clearance. *Model sketch of E-cadherin/catenin complex localization in epithelial cells and the dynamic formation of the E-cadherin/catenin complex at the basal epithelial surface during apoptotic cell clearance. In epithelial cells, E-cadherin has a basolateral localization and mediates stable cell-cell contacts by forming adherens junctions via the association with α-, β- and p120-catenins. During apoptotic cell clearance, E-cadherin localizes to the phagocyte-target interface and recruits its downstream effectors α-catenin, β-catenin and p120-catenin. At the phagocytic synapse, E-cadherin and its binding partners orchestrate actomyosin dynamics and architecture, facilitating phagocytic cup progression and uptake. Particularly, while p120-catenin downregulates Myosin II activity at the phagocytic cup, α/β-catenins function as the physical link between the complex and the actin cytoskeleton. Under E-cadherin knock down conditions, E-cadherin cannot cluster at the phagocyte-target contact site and consequently, α-, β- and p120-catenins are not recruited. This causes an hyperactivation of Myosin II at the phagocytic synapse due to the absent inhibitory effect of p120-catenin. Additionally, α/β-catenins do not longer connect the*

cytoskeleton of the phagocyte the target, being unable to direct the protrusion along the target's surface. Together, in absence of E-cadherin, phagocytic cup progression is abrogated.

• Suppl Fig. 7A. It is surprising that the authors find a significant difference given the strong overlap of the data points and the nearly identical mean cell area. I assume that this is related to the fact that the sample size is very unequal (2 fold) which reduces statistical power and affects results.

As the reviewer correctly points out, the initially analyzed sample size was rather inequal. To avoid possible misinterpretations due to this, we have now re-analyzed our sample set in the following way: for both conditions (DMSO and Blebbistatin), we selected embryos with medium/high levels of apoptotic targets and analyzed cell area of EVL cells in contact with apoptotic targets, considering a more equal sample size between conditions (EcadMO/ DMSO n = 406 cells from 12 embryos, EcadMO/Blebbistatin n = 462 cells from 12 embryos). This new analysis shows a mild but statistically significant increase in cell area in Blebbistatin-treated embryos compared to control embryos, similar to our previously presented data (Fig. R7A, Supplementary Figure 7A). In our opinion, the statistical significance is biologically relevant and, while an overlap between the data is present, their distribution also shows a significant difference (Fig. R7B). Together, these results support that inhibition of Myosin II activity via Blebbistatin in E-cadherin deficient epithelial cells rescues their phagocytic clearance defect and can partially restore tissue-level changes in epithelial architecture upon apoptotic cell interactions.

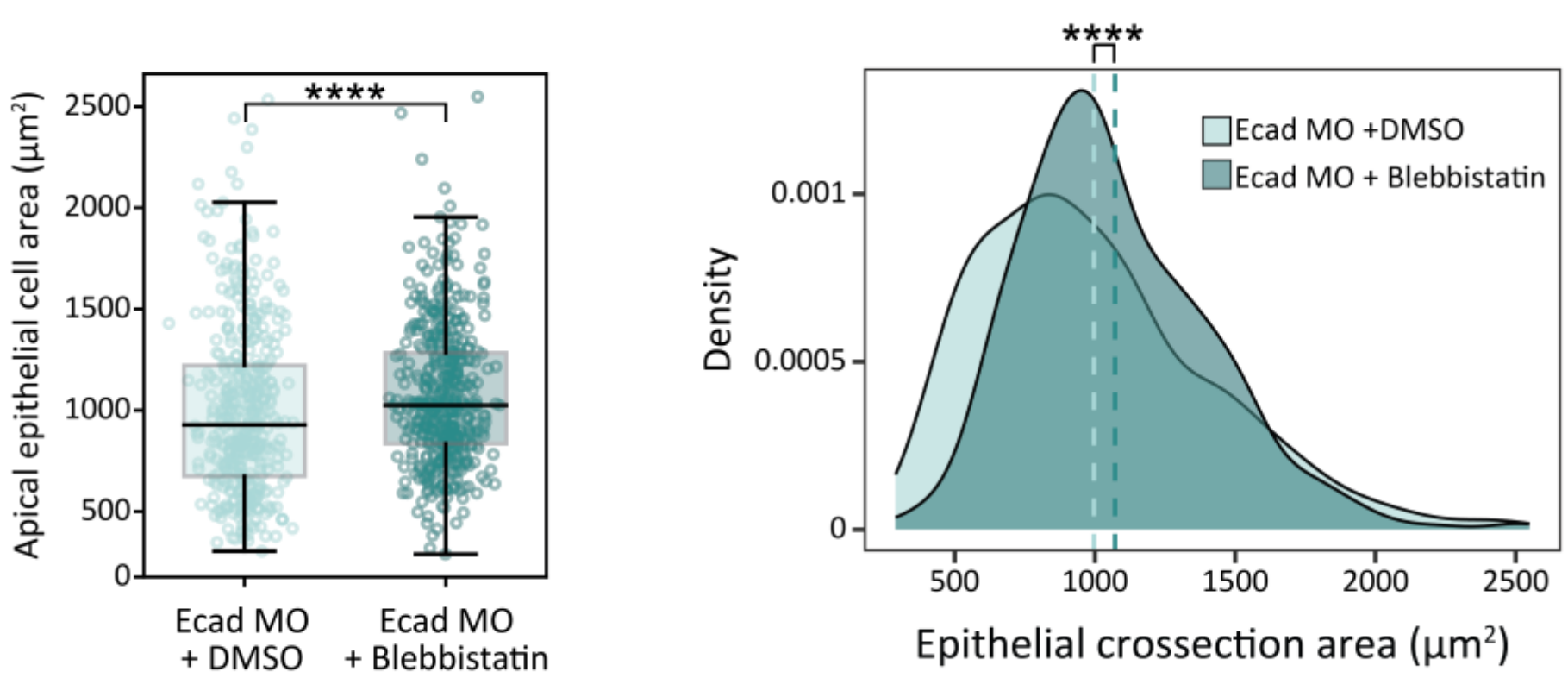

Fig R7. Myosin II downregulation partially reduces E-cadherin depleted EVL cells shrinking in contact with apoptotic targets. *Quantification of the apical area in epithelial cells in direct contact with apoptotic cells in Ecad MO embryos at 75% epiboly (7.5 hpf) incubated in DMSO (n = 406 cells from 12 embryos) or in Blebbistatin (n = 462 cells from 12 embryos) represented in a boxplot (left) and as a density distribution (right). Mann-Whitney test, p <0.0001 (A), and Kolmogorov-Smirnov test p < 0.0001 (B);N = 3.*
